# Urogenital Schistosomiasis Is Associated with an Increased Risk of *Plasmodium falciparum* Infection in Burkina Faso

**DOI:** 10.4269/ajtmh.24-0726

**Published:** 2025-05-13

**Authors:** Mireille Ouedraogo, Pytsje T. Hoekstra, Youssouf Kabore, Issa Nebie, Stan Hilt, Govert J. van Dam, Paul L. A. M. Corstjens, Fabrizio Bruschi, David Modiano, Valentina D. Mangano

**Affiliations:** ^1^ Department of Translational Research in Medicine and Surgery, University of Pisa, Pisa, Italy;; ^2^ Department of Public Health and Infectious Diseases, University of Rome La Sapienza, Rome, Italy;; ^3^ Centre National de Recherche et Formation sur le Paludisme, Ouagadougou, Burkina Faso;; ^4^ Leiden University Center for Infectious Diseases, Leiden University Medical Centre, Leiden, The Netherlands;; ^5^ Department of Cell & Chemical Biology, Leiden University Medical Center, Leiden, The Netherlands

## Abstract

There is significant overlap in the global distribution of malaria and neglected tropical diseases, with the largest health burden in Sub-Saharan Africa, where areas are co-endemic for malaria and schistosomiasis, soil-transmitted helminths, or lymphatic filariasis. Some studies suggest that helminth infections may increase susceptibility to malaria, but evidence is limited. This study investigated the association between urogenital schistosomiasis and the risk of *Plasmodium falciparum* (*P. falciparum*) parasitemia in rural Burkina Faso. A cohort of 424 subjects participated in five cross-sectional malaria surveys. Active *Schistosoma hematobium* infection was diagnosed at baseline using plasma circulating anodic antigen detection, whereas *P. falciparum* infection was diagnosed at each survey via blood smear microscopy. Longitudinal analysis assessed the association between baseline urogenital schistosomiasis and *P. falciparum* parasitemia over time, adjusting for sex, age, village, ethnicity, and hemoglobin genotype. Subjects with active urogenital schistosomiasis had a ∼ 25% increase in the cumulative incidence of *P. falciparum* infection (incidence rate ratio [IRR] = 1.26; 95% CI = 1.08–1.46; *P* = 0.004), as well as a nonsignificant trend toward higher parasite density (exponential of the β coefficient [Expβ] = 1.12; 95% CI = 0.96–1.31; *P* = 0.133) and higher odds of infection over five surveys (odds ratio [OR] = 1.79; 95% CI = 0.89–3.59; *P* = 0.104). Higher intensity of schistosomiasis increased the cumulative incidence of *P. falciparum* (IRR = 1.12; 95% CI = 1.05–1.19; *P* = 0.001) and parasite density (Expβ = 1.08; 95% CI = 1.01–1.15; *P* = 0.026), and a trend toward increased odds of infection was also observed (OR = 1.28; 95% CI = 0.91–1.80; *P* = 0.159). This study provides longitudinal evidence that urogenital schistosomiasis is associated with an increased risk of *P. falciparum* parasitaemia, highlighting the need for integrated control strategies for both diseases, particularly in school-aged children and adolescents.

## INTRODUCTION

Malaria, despite a significant decrease in both mortality and morbidity since the beginning of the millennium, still represents a major threat to global public health, with an estimated 608,000 deaths and 249 million clinical cases in 2022,^1^ along with 55.2 million disability-adjusted life years (DALYs) lost yearly, accounting for 1.9% of all-cause DALYs.^2^ Malaria is caused by *Plasmodium* protozoan parasites and is transmitted by *Anopheles* female mosquitoes in 85 countries, primarily low- and middle-income countries in tropical and subtropical climate areas. Sub-Saharan Africa (SSA) carries the largest malaria burden, with ∼95% of global deaths and cases, as well as the highest prevalence of the deadliest species, *Plasmodium falciparum* (*P. falciparum*).^1^

Together with malaria, neglected tropical diseases (NTDs) impose a disproportionate health burden on populations living in low- and middle-income countries.^3^ Neglected tropical diseases are a diverse group of diseases caused by pathogens belonging to different taxa and transmitted by various routes. They were grouped together by the WHO because of their higher prevalence in tropical and subtropical climate areas, their strong association with poverty, and the historically scarce attention they have received, with investments devoted to control not proportional to their health impact.^4^^–^^5^ Indeed, NTDs are most common among vulnerable populations in rural or impoverished urban areas and war zones, affecting 1.6 billion people worldwide^3^ and causing 9.1 million DALYs each year, which is 0.6% of the total.^2^

Helminth infections represent the largest group of NTDs and show a significant overlap with malaria in their geographic distribution at the global level.^6^ Sub-Saharan Africa carries the heaviest burden of both malaria and helminth infections,^2^ and large areas of co-endemicity exist, particularly between malaria and schistosomiasis or soil-transmitted helminths (STHs).^7^

In areas of co-endemicity, the probability of coinfection depends on the prevalence of each parasitic infection as well as its age distribution.^8^ Previous studies have indicated that school-aged children are the age group at higher risk of coinfection between plasmodia and either schistosomes or STHs, or both.^8^

It has been suggested that such coinfection could have an additive or synergistic effect on morbidity. This hypothesis is supported by the observation that anemia is worsened in children with both malaria and hookworm infection compared to those with single infections.^9^ Furthermore, hepatomegaly, splenomegaly, anorexia, malabsorption, and chronic blood loss caused by schistosomiasis or STHs contribute not only to anemia but also to micronutrient deficiency and malnutrition, which may impair the immune system and increase susceptibility to other infections, including malaria. The overall health impact of these factors is likely currently underestimated.^9^

Concomitant infection with helminths and intracellular pathogens might impact the immune response; although helminth infections typically induce type 2 and regulatory immune responses, protective responses to intracellular pathogens such as malaria require type 1 and cytotoxic immune responses.^6^ Infection with helminths might therefore modulate immune responses to malaria, potentially affecting both natural immunity and vaccine efficacy.

Indeed, some epidemiological studies suggest that infection with helminths might increase susceptibility to malaria infection. Regarding schistosomiasis, a systematic review and meta-analysis of eight studies conducted in SSA showed an increase in *P. falciparum* prevalence among *Schistosoma*-infected subjects compared with uninfected ones (odds ratio [OR] = 1.8; 95% CI = 1.4–2.3).^10^ A similar systematic review and meta-analysis of seven studies conducted in SSA showed an increase in *P. falciparum* prevalence among STH-infected subjects compared with uninfected ones (OR = 1.4; 95% CI = 1.05–1.87).^11^ However, in both instances, the reviews included studies based on single cross-sectional surveys, thereby providing limited evidence. A more recent meta-analysis of studies conducted in SSA, South America, and Southeast Asia, with similar limitations, did not reach conclusive evidence regarding the association of schistosomiasis with *P. falciparum* prevalence (14 studies; OR = 0.65; 95% CI = 0.37–1.16) but confirmed an increased *P. falciparum* prevalence in STH-infected subjects (24 studies; OR = 1.3; 95% CI = 1.03–1.65).^12^

To gain more robust evidence on the effect of helminth NTDs on susceptibility to malaria, the present investigation assessed the association between helminth infection and the prospective risk of *P. falciparum* parasitaemia, leveraging a malaria longitudinal study previously conducted among Fulani, Rimaibe, and Mossi communities living in rural villages of central Burkina Faso.^13^ In particular, the investigation focused on urogenital schistosomiasis because *Schistosoma hematobium* (*S. hematobium*) has been shown to be the most prevalent helminth infection in the study area.^14^

## MATERIALS AND METHODS

### Study area.

This study was conducted in two rural villages of Burkina Faso, located northeast of the capital Ouagadougou in the Ziniare District of the Plateau Central Region: Barkoumbilen, inhabited by Mossi and Rimaibe communities, and Barkoundouba, inhabited by Fulani and Rimaibe communities. Malaria transmission is hyperendemic and seasonal, with a rainy season from June to October. The entomological inoculation rates, estimated at ∼100–200 infective bites per person per year, are similar across the villages.^15^ Urogenital schistosomiasis is also endemic in the area,^16^^–^^17^ with a prevalence of *S. hematobium* of 29%.^17^ The Fulani have been previously reported to be less infected with *P. falciparum* and to have a different genetic background from their neighbors, whereas the Mossi and Rimaibe show comparable susceptibility to infection and are genetically similar.^15^^,^^18^^,^^19^ Therefore, they will be grouped together.

### Study design.

The entire communities were invited to participate in the study. The inclusion criteria were living in the study villages and willingness to participate, whereas the exclusion criteria included clinical conditions requiring urgent care. The study used a repeated cross-sectional design: five malariological surveys were conducted at the beginning (August) and end (November/December) of the 2007 and 2008 high malaria transmission seasons, as well as in the middle of the intervening dry low transmission season (March 2008).^13^ During each survey, demographic information was collected, and a team of physicians examined participants for clinical signs, measured axillary body temperature, and prepared blood slides from finger pricks to be subsequently analyzed in the laboratory for the microscopic diagnosis of malaria. Subjects exhibiting fever (temperature ≥37.5°C) were treated presumptively for malaria with artemether-lumefantrine (Coartem) according to the manufacturer’s dosage recommendations because parasitological confirmation would have significantly delayed treatment. During the first survey, when a subject entered the study, a 2 mL venous blood sample was collected in ethylenediaminetetraacetic acid tubes for DNA isolation, hemoglobin genotyping,^13^ plasma separation, and serological assays. Plasma was separated by centrifugation (3 minutes at 2,000 rpm), aliquoted, and stored at −80°C until assays were performed.

Active infection with *S. hematobium* was retrospectively assessed at the first survey (baseline) by measuring circulating anodic antigen (CAA) in plasma among subjects who participated in all five malaria cross-sectional surveys and for whom plasma specimens were available (*n* = 424). Because the diagnosis of urogenital schistosomiasis was retrospective, it was not possible to offer treatment to individuals with active infection.

Analysis of the first cross-sectional data was conducted to describe the prevalence and intensity of urogenital schistosomiasis at baseline and to assess variation according to demographic factors. Longitudinal analysis of repeated cross-sectional data was performed to assess the association between urogenital schistosomiasis at baseline and the prospective risk of *P. falciparum* infection.

### Malaria microscopy.

*Plasmodium falciparum* asexual parasitemia was diagnosed microscopically.^13^ Blood slides with thick and thin blood smears were prepared and stained with Giemsa stain according to standard procedures and were read independently by two skilled microscopists. The *Plasmodium* species was identified on the thin blood smear. Readers examined 100 microscopic fields (corresponding to 0.25 µL of blood) from the thick blood smear; parasite counts were converted to numbers of parasites per microliter of blood (assuming a standard count of 8,000/µL), and the mean density from the two readings was used. A third reader was involved when the two readers disagreed about positivity or when estimated densities differed by >30%. In these cases, the mean of the two closest density readings was used.

### Schistosoma circulating anodic antigen.

The up-converting particle lateral flow assay was conducted at Leiden University Medical Center, the Netherlands, to detect CAA in plasma samples, as previously described (SCAA20, wet test format).^17^^,^^20^ A dilution series of a sample with a known CAA concentration (pg/mL) was included and used as a standard curve to quantify CAA concentrations and validate the threshold of the assay (10 pg/mL). Circulating anodic antigen levels were defined on the basis of concentration ranges as follows: level 0 = 0–9 pg/mL; level 1 = 10–99 pg/mL; level 2 = 100–999 pg/mL; level 3 = 1,000–9,999 pg/mL; level 4 ≥10,000 pg/mL. It was previously shown that the area is endemic for *S. hematobium* and not *S. mansoni*^17^; therefore, CAA was used as a marker of active urogenital schistosomiasis.

## STATISTICAL ANALYSES

The prevalence of urogenital schistosomiasis in the first survey was estimated by computing the proportion (%) of CAA-positive subjects, along with its 95% CI, as shown in bar plots. An analysis of associations with age group (0–4, 5–9, 10–19, 20–39, 40–80 years), sex (males, females), ethnicity (Fulani, Mossi-Rimaibe), and village (Barkoundouba, Barkoumbilen) was conducted using multivariate logistic regression, reporting the OR with its 95% CI and the *P*-value.

The intensity of urogenital schistosomiasis in the first survey was estimated by computing the mean and standard deviation of CAA concentration (pg/mL) among positive subjects and presenting boxplots. An analysis of the association with the same demographic factors as mentioned above was conducted using multivariate linear regression, reporting the β coefficient along with its 95% CI and the *P*-value.

A longitudinal analysis of repeated cross-sectional data was conducted to assess the association between active urogenital schistosomiasis at baseline and the prospective risk of *P. falciparum* infection, regardless of whether parasitemia was associated with fever. The exposure variables were *S. hematobium* CAA positivity (yes/no) or CAA levels (0 = negative, 1 = low, 2 = moderate, 3 = high, 4 = very high) at baseline. The outcome variables included the odds of *P. falciparum* infection (i.e., the odds of being infected at least once; yes/no), the cumulative incidence of *P. falciparum* infection (number of infections; from 0 to 5), and the mean *P. falciparum* parasite density (i.e., individual mean of log-transformed par/µL values over five measurements) during the study period. An association analysis between exposure and outcome variables was conducted using logistic, Poisson, and linear regression models, as appropriate, reporting the risk ratio with its 95% CI and *P*-value. All regression models included age groups, sex, ethnicity, village of residence, and hemoglobin genotype (AA, AS, AC, CC, SC) as covariates.

Statistical differences were considered significant at *P* ≤0.05. All analyses were conducted using the statistical software package Stata/IC 13.0 (StataCorp LP, College Station, TX).

## RESULTS

### Prevalence and intensity of urogenital schistosomiasis at baseline.

The prevalence and intensity of urogenital schistosomiasis in the study area were assessed by measuring *S. hematobium* CAA in plasma specimens collected during the first of five cross-sectional surveys.

In the overall study population, the proportion of subjects positive for CAA (CAA positivity) was 28.3% (120/424). An analysis of associations with demographic factors (Table 1) showed that CAA positivity was lowest in children aged 1 to 4 years, increased in children aged 5 to 9 years, reached a maximum in adolescents aged 10 to 19 years, and decreased in adults older than 20 years. Additionally, CAA positivity was comparable in females and males. Finally, CAA positivity was higher in Barkoumbilen than in Barkoundouba, whereas no differences were observed between sympatric ethnic groups. Apparent differences in the proportion of positives between ethnic groups were actually explained by differences in village of residence and were therefore not statistically significant. When comparing ethnic groups within each village, no differences were observed between Fulani and Rimaibe in Barkoundouba (Fulani = 12.4%; Rimaibe = 12.9%; OR = 1.05; 95% CI = 0.32–3.41; *P* = 0.939) or between Mossi and Rimaibe in Barkoumbilen (Mossi = 41.4%; Rimaibe = 32.3%; OR = 0.67; 95% CI = 0.41–1.11; *P* = 0.122).

**Table 1:** Proportion of circulating anodic antigen-positive subjects according to demographic factors

Subject Characteristics		Total (*N*)	CAA Positive Subjects				Association with CAA Positivity			
			Pos (*n*)	Pos (%)	LCL (%)	UCL (%)	OR	LCL	UCL	*P*-Value
Age group (years)	1–4 (ref)	93	4	4.3	1.6	11.0	–	–	–	–
	5–9	107	32	29.9	21.9	39.3	12.9	4.2	38.9	0.000
	10–19	99	58	58.6	48.6	67.9	54.2	17.4	168.6	0.000
	20–39	71	14	19.7	12.0	30.7	6.7	2.0	22.4	0.002
	40–80	54	12	22.2	13.0	35.4	7.5	2.2	25.6	0.001
Sex	Males (ref)	251	54	31.2	24.7	38.5	–	–	–	–
	Females	173	66	26.3	21.2	32.1	0.7	0.4	1.2	0.218
Village	Barkoundouba (ref)	152	19	12.5	8.1	18.8	–	–	–	–
	Barkoumbilen	272	101	37.1	31.6	43.1	7.3	2.3	23.7	0.001
Ethnicity	Fulani (ref)	121	15	12.4	7.6	19.6	–	–	–	–
	Mossi-Rimaibe	303	105	34.7	29.5	40.2	1.0	0.3	3.4	0.958
Total (*N*)		424	120	28.3	24.2	32.8	–	–	–	–

CAA = circulating anodic antigen; LCL = 95% lower confidence limit; OR = odds ratio; pos = positive subjects; ref = reference group; UCL = 95% upper confidence limit. Number (*n*) and proportion (%) of CAA positive subjects according to demographic characteristics of study participants and results of multivariate logistic regression analysis (OR and *P*-value) investigating the association of demographic factors with CAA positivity.

Among positive subjects, the mean concentration of CAA was 1,706 pg/mL, corresponding to the third of four levels, indicating high infection intensity. The variation in CAA concentration (pg/ml) among positive subjects was also assessed according to age group, sex, village, and ethnicity (Table 2), but no significant differences were observed.

**Table 2:** Concentration of circulating anodic antigen-among positive subjects according to demographic factors

Subject Characteristics		Positive Subjects (*n*)	CAA Concentration (pg/mL)		Association with CAA Concentration			
			Mean	SD	βcoeff	LCL	UCL	*P*-Value
Age-group (years old)	1–4 (ref)	4	1,091.6	597.3	–	–	–	–
	5–9	32	1,702.8	464.0	714.6	−2,019.7	3,448.9	0.606
	10–19	58	2,302.0	401.4	1,405.4	−1,268.3	4,079.1	0.300
	20–39	14	667.9	345.7	−338.5	−3,294.5	2,617.6	0.821
	40–80	12	247.5	144.9	−794.3	−3,857.6	2,269.0	0.608
Sex	Males (ref)	54	1,702.4	331.2	–	–	–	–
	Females	66	1,708.5	349.5	279.5	−697.6	1,256.7	0.572
Village	Barkoundouba (ref)	19	820.8	389.1	–	–	–	–
	Barkoumbilen	101	1,872.3	275.8	385.5	−2,503.9	3,274.8	0.792
Ethnicity	Fulani (ref)	15	1,014.2	483.6	–	–	–	–
	Mossi-Rimaibe	105	1,804.6	267.3	717.4	−2,485.9	3,920.6	0.658
Total		120	1,705.8	242.2	–	–	–	–

βcoeff = β coefficient; CAA = circulating anodic antigen; LCL = 95% lower confidence limit; ref = reference group; UCL = 95% upper confidence limit. Mean concentration (pg/mL) of CAA among positive subjects according to demographic characteristics of study participants and results of multivariate linear regression analysis (βcoeff and *P*-value) investigating the association of demographic factors with CAA positivity.

### Coinfection between urogenital schistosomiasis and malaria at baseline.

The prevalence of urogenital schistosomiasis measured during the first survey was compared with that of *P. falciparum* parasitemia, and the prevalence of coinfection was assessed in the overall population, as well as by demographic factors (Table 3).

**Table 3:** Prevalence of *Plasmodium falciparum*, *Schistosoma hematobium*, and coinfection with the two parasites

Subject Characteristics		*P. falciparum*			*S. hematobium*			Coinfection		
		Pos (%)	LCL (%)	UCL (%)	Pos (%)	LCL (%)	UCL (%)	Pos (%)	LCL (%)	UCL (%)
Age group (years old)	1–4 (ref)	60.2	49.9	69.7	4.3	1.6	11.0	4.3	1.6	11.0
	5–9	67.3	57.8	75.6	29.9	21.9	39.3	19.6	13.1	28.3
	10–19	55.6	45.6	65.1	58.6	48.6	67.9	36.4	27.4	46.3
	20–39	23.9	15.4	35.3	19.7	12.0	30.7	5.6	2.1	14.2
	40–80	18.5	10.2	31.3	22.2	13.0	35.4	3.7	0.9	13.8
Sex	Males (ref)	57.8	50.3	65.0	31.2	24.7	38.5	19.7	14.4	26.3
	Females	43.8	37.8	50.1	26.3	21.2	32.1	14.3	10.5	19.3
Village	Barkoundouba (ref)	40.8	33.2	48.8	12.5	8.1	18.8	5.3	2.6	10.2
	Barkoumbilen	54.4	48.4	60.3	37.1	31.6	43.1	22.8	18.2	28.2
Ethnicity	Fulani (ref)	38.0	29.8	47.0	12.4	7.6	19.6	6.6	3.3	12.7
	Mossi-Rimaibe	54.1	48.5	59.7	34.7	29.5	40.2	20.5	16.3	25.4
Total		49.5	44.8	54.3	28.3	24.2	32.8	15.8	12.6	19.6

LCL = 95% lower confidence limit; *P. falciparum* = *Plasmodium falciparum*; pos = proportion of positive subjects; ref = reference group; *S. hematobium* = *Schistosoma hematobium*; UCL = 95% upper confidence limit. Proportion of subjects infected with *P. falciparum*, with *S. Hematobium*, or with both parasites according to subjects’ demographic characteristics.

The prevalence of malaria infection in the overall population was 49.5% (210/424). The proportion of infected subjects was highest among children below 10 years of age and showed a slight decrease in adolescents aged 10–19 years and a sharper decrease in adults older than 20 years.

**Figure 1.**
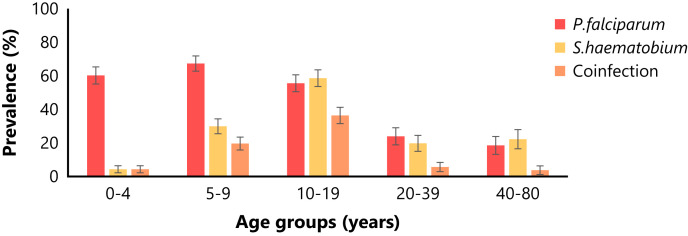
Age-group prevalence of *Plasmodium falciparum* (*P. falciparum*), *Schistosoma hematobium* (*S. hematobium*), and coinfection with both parasites. Prevalence (%) of *P. falciparum* and *S. hematobium* infections, as well as the coinfection with both parasites, stratified by age group. Error bars represent the standard error of the proportion. Age groups: 0–4 years old (*n* = 93), 5–9 years old (*n* = 107), 10–19 years old (*n* = 99), 20–39 years old (*n* = 71), 40–80 years old (*n* = 54).

The prevalence of coinfection between *S. hematobium* and *P. falciparum* in the overall population was 15.8% (67/424). The proportion of coinfected subjects was lowest in children aged 1–4 years, increased in children aged 5–9 years, and reached a maximum in adolescents aged 10–19 years, whereas it decreased in adults older than 20 years (Figure 1). A similar proportion of coinfected subjects was observed in females and males. Coinfection was more frequent in Barkoumbilen than in Barkoundouba, as well as in Mossi-Rimaibe compared with Fulani.

### Association of urogenital schistosomiasis with risk of malaria infection.

Finally, to assess whether active urogenital schistosomiasis affects the prospective risk of *P. falciparum* parasitemia among study populations, a longitudinal analysis of repeated cross-sectional data has been performed.

The association between either the presence (yes/no) or the level (i.e., concentration range) of *S. hematobium* CAA at baseline and the odds of being infected at least once with *P. falciparum* over time, the cumulative incidence of infection, and the mean parasite density has been assessed using regression models adjusted for age group, sex, village, ethnicity, and hemoglobin genotype.

Multivariate regression analysis investigating the association of both *S. hematobium* infection (Table 4) and intensity (Table 5) showed that the covariates age group, sex, ethnicity, and hemoglobin genotype affected the risk of *P. falciparum* parasitemia. In particular, susceptibility to malaria infection was significantly reduced in older age groups and in subjects carrying hemoglobin S or C, whereas it was significantly increased in subjects of the Mossi-Rimaibé ethnicity.

**Table 4:** Results of multivariate regression association analysis between *Schistosoma hematobium* circulating anodic antigen positivity and *Plasmodium falciparum* parasitemia

Independent Variable	Odds of *P. falciparum* Infection			Incidence of *P. falciparum* Infection			Mean *P. falciparum* Density		
	OR	95% CI	*P*-Value	IRR	95% CI	*P*-Value	Exp(β)	95% CI	*P*-Value
Age group	0.32	0.25–0.43	0.000	0.75	0.71–0.79	0.000	0.70	0.67–0.74	0.000
Sex	0.71	0.37–1.38	0.311	0.90	0.78–1.03	0.110	0.84	0.73–0.96	0.013
Village	1.01	0.99–1.03	0.167	1.03	1.01–1.05	0.024	1.03	1.01–1.06	0.011
Ethnicity	17.48	3.74–81.78	0.000	2.00	1.54–2.61	0.000	2.37	1.81–3.11	0.000
*HBB* genotype	0.53	0.36–0.79	0.002	0.92	0.83–1.02	0.106	0.90	0.82–0.99	0.036
CAA positivity	1.79	0.89–3.59	0.104	1.26	1.08–1.46	0.004	1.12	0.96–1.31	0.133

CAA = circulating anodic antigen; Exp(β) = exponential of the β coefficient; *HBB* = hemoglobin beta chain locus; IRR = incidence rate ratio; OR = odds ratio; *P. falciparum* = *Plasmodium falciparum*. Results of multivariate regression models assessing the association between CAA positivity (yes/no) and 1) the odds of being infected with *P. falciparum* (yes/no; logistic regression), 2) the incidence of *P. falciparum* infections (0–5 infections; Poisson regression), and 3) the mean *P. falciparum* density (mean of log-transformed par/mcl values; linear regression) over five measurements. Regression model included demographic characteristics and hemoglobin genotype as covariates.

**Table 5:** Results of multivariate regression association analysis between *Schistosoma hematobium* circulating anodic antigen levels and *Plasmodium falciparum* parasitemia

Independent Variable	Odds of *P. falciparum* Infection			Incidence of *P. falciparum* Infection			Mean *P. falciparum* Density		
	OR	95% CI	*P*-Value	IRR	95% CI	*P*-Value	Exp(β)	95% CI	*P*-Value
Age group	0.33	0.25–0.44	0.000	0.75	0.71–0.79	0.000	0.70	0.67–0.74	0.000
Sex	0.70	0.36–1.35	0.287	0.89	0.78–1.02	0.102	0.84	0.73–0.96	0.013
Village	1.01	1.00–1.03	0.155	1.03	1.01–1.05	0.019	1.04	1.01–1.06	0.008
Ethnicity	18.14	3.88–84.89	0.000	2.01	1.54–2.61	0.000	2.37	1.81–3.11	0.000
*HBB* genotype	0.53	0.35–0.78	0.002	0.91	0.82–1.02	0.094	0.90	0.82–0.99	0.032
CAA level	1.28	0.91–1.80	0.159	1.12	1.05–1.19	0.001	1.08	1.01–1.15	0.026

CAA = circulating anodic antigen; Exp(β) = exponential of the β coefficient; *HBB* = hemoglobin beta chain locus; IR = incidence rate ratio; OR = odds ratio; *P. falciparum* = *Plasmodium falciparum*. Results of multivariate regression models assessing the association between CAA level (0, 1, 2, 3, 4) and 1) the odds of being infected with *P. falciparum* (yes/no; logistic regression), 2) the incidence of *P. falciparum* infection (0–5 infections; Poisson regression), and 3) the mean *P. falciparum* density (mean of log-transformed par/mcl values; linear regression) over five measurements. Regression model included demographic characteristics and hemoglobin genotype as covariates.

**Figure 2.**
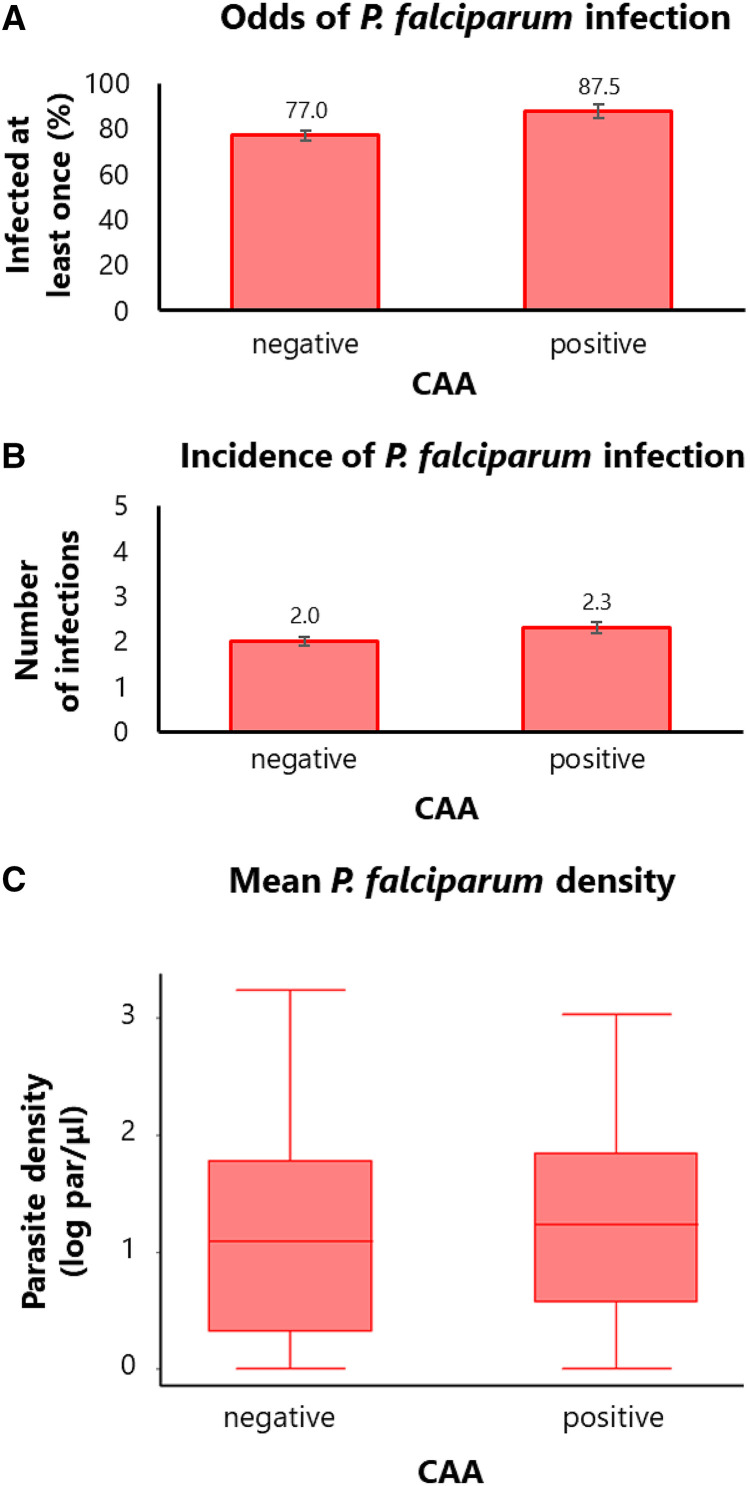
Longitudinal malaria outcomes according to the presence of *Schistosoma hematobium* (*S. hematobium*) infection. Comparison of longitudinal malaria outcomes between subjects without (circulating anodic antigen [CAA]-negative) and with active *S. hematobium* infection (CAA-positive) at baseline. (**A**) Bar plot showing the proportion of subjects infected at least once with *Plasmodium falciparum* (*P. falciparum*) over the five cross-sectional surveys (the proportion describes the probability of infection, not the odds, but it is presented as a proxy for easier interpretation)*.* (**B**) Bar plot showing the number of *P. falciparum* infections over the five cross-sectional surveys (cumulative incidence of infection). (**C**) Box plot showing the distribution of *P. falciparum* densities averaged over the five cross-sectional surveys (mean parasite density).

**Figure 3.**
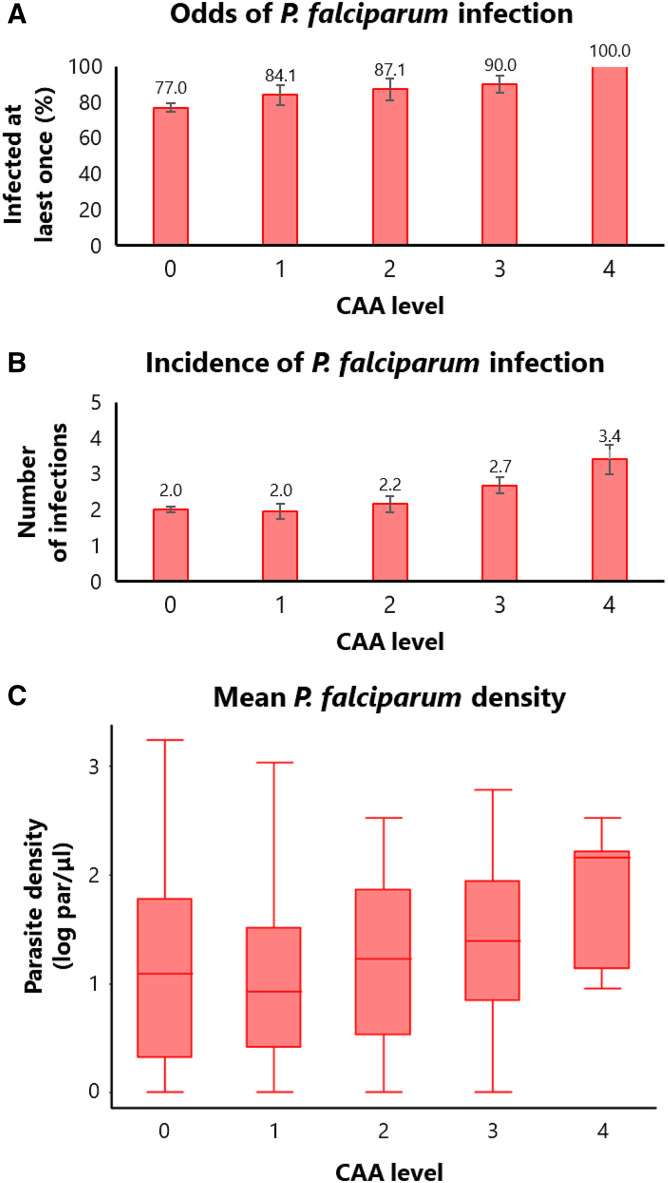
Longitudinal malaria outcomes according to the intensity of *Schistosoma hematobium* (*S. hematobium*) infection. Comparison of longitudinal malaria outcomes among subjects with different intensities of *S. hematobium* infection (circulating anodic antigen levels). (**A**) Bar plot showing the proportion of subjects infected at least once with *Plasmodium falciparum* (*P. falciparum*) over the five cross-sectional surveys (the proportion describes the probability of infection, not the odds, but it is presented as a proxy for easier interpretation)*.* (**B**) Bar plot showing the number of *P. falciparum* infections over the five cross-sectional surveys (cumulative incidence of infection). (**C**) Box plot showing the distribution of *P. falciparum* densities averaged over the five cross-sectional surveys (mean parasite density).

The results of the association analysis (Table 4) showed that CAA positivity increased the odds of being infected with *P. falciparum* during the study period by 79%, although the association did not reach statistical significance (OR = 1.79; 95% CI = 0.89–3.59; *P*-value = 0.104). The proportion of subjects who were infected at least once with *P. falciparum* during the study period was 87.5% among CAA positive subjects compared with 77% among CAA negative subjects (Figure 2A; Supplemental Table 1). On the other hand, CAA positivity significantly increased the cumulative incidence of *P. falciparum* by 26% (RR = 1.26; 95% CI = 1.08–1.46; *P*-value = 0.004). The cumulative incidence (number of infections per individual during the study period) was 2.3 among CAA positive subjects compared with 2.0 among CAA negative subjects (Figure 2B; Supplemental Table 2). Furthermore, a trend of increase in mean parasite density (mean of individual parasite densities during the study period) among CAA positive subjects was observed, although it was not significant (Expβ = 1.12; 95% CI = 0.96–1.31; *P*-value = 0.133). The distribution of parasite densities was higher among CAA positive subjects (median = 16.0 par/µL; interquartile range = 2.7–69.0 par/µL) than among CAA negative subjects (median = 11.3 par/µL; interquartile range = 1.0–59.3 par/µL; Figure 3C; Supplemental Table 3).

Similarly, the results of the association analysis (Table 5) showed that higher CAA levels increased the odds of being infected with *P. falciparum* during the study period by 28%, although the association did not reach statistical significance (OR = 1.28; 95% CI = 0.91–1.80; *P*-value = 0.159). The proportion of subjects who were infected at least once with *P. falciparum* during the study period increased with increasing CAA levels, from 84% at the lowest CAA concentration range to 100% at the highest CAA concentration range (Figure 3A; Supplemental Table 1). Additionally, higher CAA levels significantly increased the cumulative incidence of *P. falciparum* by 12% (incidence rate ratio [IRR] = 1.12; 95% CI = 1.08–1.46; *P*-value = 0.001). The number of infections during the study period increased from 1.95 at the lowest CAA concentration range to 3.4 at the highest CAA concentration range (Figure 3B; Supplemental Table 2). Finally, a significant increase in mean parasite density was observed (Expβ = 1.08; 95% CI = 1.01–1.15; *P*-value = 0.026). The distribution of mean parasite densities was higher at elevated CAA levels, increasing from the lowest (median = 7.4 par/µL; interquartile range = 1.5–31.6 par/µL) to the highest (median = 142.7 par/µL; interquartile range = 10.2–246.3 par/µL) CAA concentration range (Figure 3C; Supplemental Table 3).

## DISCUSSION

The observed prevalence of urogenital schistosomiasis, as diagnosed by the presence of CAA in plasma, was 28.3% in the study area of the Plateau Central region of Burkina Faso in 2007. Marked differences in prevalence were observed between the two study villages, highlighting how risk factors for schistosomiasis can vary substantially even on a microgeographic scale. This heterogeneity is known to be mostly driven by the distribution of intermediate host snails and human–water contact patterns, resulting in a focal distribution.^21^^–^^22^ As expected, prevalence increased sharply in school-aged children and adolescents^23^ but remained high in adults, who could contribute to sustained transmission. A high intensity of infection was observed, with no significant variation according to demographic factors.

It must be noted that the prevalence and intensity of urogenital schistosomiasis assessed in this study do not reflect the current epidemiological situation, given the retrospective nature of the investigation, which occurred 17 years ago. A national program targeting school-aged children with drugs against schistosomiasis and STHs was implemented in Burkina Faso starting in 2004–2005.^24^ Biennial mass drug administration (MDA) of praziquantel (600 mg) to children aged 5 to 15 years began in 2004 in four regions considered hyper-endemic for schistosomiasis, and in 2005, it spread to the remaining nine meso-endemic regions. In the study region (Plateau Central), a single round of MDA was performed in 2005,^24^ 2 years before the baseline survey of the present study in 2007. The estimated coverage (% of eligible children reached by MDA) in the study area (Ziniare district) was 80%.^25^ A substantial reduction in prevalence (−83%) and intensity (−93%) of *S. hematobium* was observed after 2 years of a single round of MDA in a longitudinal study conducted in the hyperendemic regions.^26^^–^^27^ A similar observation could be reasonably expected for the meso-endemic regions, including the study area; therefore, the observed prevalence and intensity should be interpreted as the result of a reduction due to one round of MDA. Additionally, both prevalence and intensity should be significantly lower at present compared with what was assessed in this study, thanks to further rounds of MDA.^25^ In fact, Lai and collaborators have estimated, through a systematic review of survey data and geostatistical modeling, that the population prevalence of *S. hematobium* infection was 22.6% in 2012 in Burkina Faso.^15^ Similarly, the malaria outcomes assessed in this study do not reflect the current epidemiological situation.

Coinfection between *S. hematobium* and *P. falciparum* was observed in 15.8% of subjects in the overall study population, with the highest rates among children aged 5–9 years (19.6%) and adolescents aged 10–19 years (36.4%). This observation confirms previous reports and estimates based on the age distribution of the two parasitic infections^9^^,^^28^ and indicates that this age range should be the primary target of integrated control measures.

**Figure 4.**
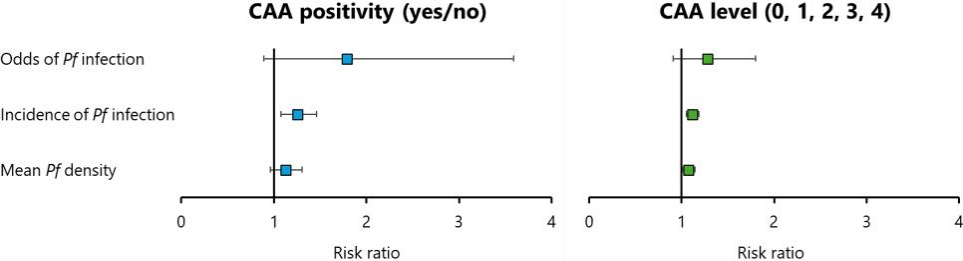
Overall results of the longitudinal association analysis between urogenital schistosomiasis at baseline and the prospective risk of *Plasmodium falciparum* parasitemia. Forest plots illustrating the results of the multivariate regression analysis of the association between circulating anodic antigen (CAA) positivity (left panel) or CAA level (right panel) and malaria longitudinal outcomes. Squares represent the RR, and whiskers represent the 95% CI of the RR.

The results of the multivariate regression analysis showed that the risk of *P. falciparum* infection was higher in subjects with urogenital schistosomiasis for the three investigated malaria outcomes, with a significant difference for the incidence of infection and a trend in the same direction for the other two outcomes. Additionally, the risk of *P. falciparum* infection increased with higher intensities of urogenital schistosomiasis, demonstrating a linear relationship and showing significant differences for the incidence of infection and the mean parasite density, as well as a trend in the same direction for the odds of infection. The results of the longitudinal association analysis are therefore coherent, and despite the limited sample size and statistical power, they indicate that active infection with *S. hematobium* and higher infection intensity with this trematode increased the risk of *P. falciparum* parasitemia in our study population (Figure 4). To the best of our knowledge, only one longitudinal study has previously evaluated the association of schistosomiasis with the prospective risk of malaria infection.^29^ The study, conducted in Gabon, revealed an increased incidence of *P. falciparum* infection among school-aged children with urogenital schistosomiasis (IRR = 1.42; 95% CI = 1.14–1.77; *P* = 0.002).^29^ The results presented here therefore confirm the findings of a previous longitudinal study conducted in a different SSA country, collectively providing the most robust evidence available to date.

In view of the literature, the increased risk of *P. falciparum* parasitemia among subjects with urogenital schistosomiasis could be interpreted as a higher susceptibility to malaria resulting from either a general impairment of the immune system or poorer protective immune responses against *Plasmodium* parasites, or both, caused by chronic *S. haematobium* infection.^6^^,^^8^^,^^9^ The results of the present investigation suggest that the health impact of schistosomiasis is likely currently underestimated because it does not account for a potential effect on malaria susceptibility.

Moreover, the results highlight the importance of implementing integrated control strategies against these two parasitic diseases.^1^^,^^30^ The WHO recommends an integrated approach for the control and elimination of NTDs to achieve success and sustainability.^31^ Sharing delivery platforms for diseases with similar epidemiology, such as schistosomiasis and malaria, offers a significant opportunity.^32^ For example, praziquantel could be delivered to school-aged children alongside malaria chemoprevention^33^ just before the malaria transmission season. This integrated approach would not only be more sustainable but also more effective compared with separate initiatives.

## CONCLUSION

In conclusion, this study has generated new evidence regarding the effect of urogenital schistosomiasis on susceptibility to malaria. Despite limitations in sample size and statistical power, an increase in the prospective risk of *P. falciparum* parasitemia has been observed among subjects with active urogenital schistosomiasis and in subjects with higher infection intensity. This evidence calls for the implementation of integrated control measures against schistosomiasis and malaria, particularly among school-aged children and adolescents, who are at higher risk of coinfection and comorbidity.

## Supplemental Materials

10.4269/ajtmh.24-0726 Supplemental Materials
