# Supplementary material for: Urogenital Schistosomiasis Is Associated with an Increased Risk of Plasmodium falciparum Infection in Burkina Faso
Source: Am J Trop Med Hyg. 2025 May 13;113(1):67–75. doi: 10.4269/ajtmh.24-0726 (PMC12225574; doi:10.4269/ajtmh.24-0726)
Supplement: Supplemental Materials [file tpmd240726.SD1.pdf]

SUPPLEMENTAL INFORMATION

Supplemental Table 1. Association between CAA (positivity and levels) and the odds of *P. falciparum* infection

CAA	Infected with *Pf* at least once				Crude OR (95% CI)	Crude *P*-Value	Adjusted OR (95% CI)	Adjusted *P*-Value
	Yes	No	Total	Proportion (95%CI)				
neg	234	70	304	77.0 (71.9-81.4)	-	0.016	-	0.104
pos	105	15	120	87.5 (80.2-92.3)	2.09 (1.15-3.83)		1.79 (0.89-3.59)	

CAA level	Infected with *Pf* at least once				Crude OR (95% CI)	Crude *P*-Value	Adjusted OR (95% CI)	Adjusted *P*-Value
	Yes	No	Total	Proportion (95%CI)				
neg	234	70	304	77.0 (71.9-81.4)	-	0.012	-	0.159
low	37	7	44	84.1 (70.0-92.3)	1.45 (1.09-1.95)		1.28 (0.91-1.80)	
moderate	27	4	31	87.1 (69.8-95.2)				
high	36	4	40	90.0 (75.9-96.3)				
very high	5	0	5	100 (.)				

Results of logistic regression analysis investigating the association between CAA positivity (panel a) or CAA levels (panel b) with the odds of *P. falciparum* infection (i.e. odds of being infected at least once) during the study period. The number and the proportion of *P. falciparum* infected subjects according to CAA positivity/levels are shown, together with the Odds Ratio (OR) and *P*-value resulting from univariate and multivariate logistic regression analysis.

Supplemental Table 2. Association between CAA (positivity and levels) and incidence of *P. falciparum* infection

CAA	Number of Pf infections							N participants	Cumulative Incidence	Crude RR (95% CI)	Crude *P*-Value	Adjusted RR (95% CI)	Adjusted *P*-Value
	0	1	2	3	4	5	Total						
neg	70	50	64	64	41	15	609	304	2.00	-	<0.001	-	0.004
pos	15	20	28	32	20	5	277	120	2.31	2.00 (1.83-2.17)		1.26 (1.08-1.46)	

CAA level	Number of Pf infections							N participants	Cumulative Incidence	Crude RR (95% CI)	Crude *P*-Value	Adjusted RR (95% CI)	Adjusted *P*-Value
	0	1	2	3	4	5	Total						
neg	70	50	64	64	41	15	609	304	2.00	-	<0.001	-	<0.001
low	7	10	13	8	4	2	86	44	1.95	1.97 (1.83-2.13)		1.12 (1.05-1.19)	
moderate	4	5	9	8	5	0	67	31	2.16				
high	4	5	5	15	8	3	107	40	2.68				
very high	0	0	1	1	3	0	17	5	3.40				

Results of Poisson regression analysis investigating the association between CAA positivity (panel a) or CAA levels (panel b) with the cumulative incidence of *P. falciparum* infection (i.e. number of infections) during the study period. The number of infections, the number of study participants and the cumulative incidence according to CAA positivity/levels are shown, together with the Risk Ratio (IR) and *P*-value resulting from univariate and multivariate Poisson regression analysis.

Supplemental Table 3. Association between CAA (positivity and levels) and mean *P. falciparum* density

CAA	Pf parasite density (par/mcl)			Crude Expβ (95% CI)	Crude *P*-Value	Adjusted Expβ (95% CI)	Adjusted *P*-Value
	Median	IQR	N				
neg	11.3	1.0-59.3	304	-	0.309	-	
pos	16.0	2.7-69.0	120	1.10 (0.91-1.33)		1.12 (0.96-1.31)	0.137

CAA level	Pf parasite density (par/mcl)			Crude Expβ (95% CI)	Crude *P*-Value	Adjusted Expβ (95% CI)	Adjusted *P*-Value
	Median	IQR	N				
neg	11.3	1.0-59.3	304.0	-	0.044	-	0.027
low	7.4	1.5-31.6	44.0	1.08 (1.00-1.17)		1.08 (1.01-1.15)	
moderate	15.9	2.3-72.2	31.0				
high	23.6	5.2-88.3	40.0				
very high	142.7	10.2-246.3	5.0				

Results of linear regression analysis investigating the association between CAA positivity (panel a) or CAA levels (panel b) with the mean *P. falciparum* parasite density (i.e. individual mean of log-transformed parasite densities) during the study period. The median and interquartile range (IQR), according to CAA positivity/levels are shown, together with the exponentiated Beta coefficient (Exp**β**) and *P*-value resulting from univariate and multivariate linear regression analysis.
